# Simultaneous Detection of Bovine Rotavirus, Bovine Parvovirus, and Bovine Viral Diarrhea Virus Using a Gold Nanoparticle-Assisted PCR Assay With a Dual-Priming Oligonucleotide System

**DOI:** 10.3389/fmicb.2019.02884

**Published:** 2019-12-12

**Authors:** Mengmeng Wang, Yue Yan, Ruichong Wang, Li Wang, Han Zhou, Yijing Li, Lijie Tang, Yigang Xu, Yanping Jiang, Wen Cui, Xinyuan Qiao

**Affiliations:** ^1^Heilongjiang Key Laboratory for Animal Disease Control and Pharmaceutical Development, Department of Preventive Veterinary Medicine, College of Veterinary Medicine, Northeast Agricultural University, Harbin, China; ^2^Department for Radiological Protection, Heilongjiang Province Center for Disease Control and Prevention, Harbin, China

**Keywords:** dual-priming oligonucleotide, nanoparticle-assisted PCR assay, bovine rotavirus, bovine parvovirus, bovine viral diarrhea virus

## Abstract

Bovine rotavirus (BRV), bovine parvovirus (BPV), and bovine viral diarrhea virus (BVDV) are the pathogens that cause diarrhea primarily in newborn calves. A mixed infection of BRV, BPV, and BVDV makes clinical diagnosis difficult. In this study, we designed dual-priming oligonucleotide (DPO) primers the VP6 gene of BRV, VP2 gene of BPV, and 5′UTR gene of BVDV and synthesized gold nanoparticles (GNPs) with an average diameter of 10 nm. We combined the DPOs with the GNPs to develop a DPO-nanoPCR assay for detecting BRV, BPV, and BVDV. The annealing temperature, primer concentration, and GNP concentration were optimized for this assay. Compared to a conventional PCR assay, the DPO-nanoPCR assay allowed the use of a wider range of annealing temperatures (41–65°C) to effectively amplify target genes. PCR amplification was the most efficient at 56.2°C using conventional primers. The optimal volume of all the primers (10 μM) was 1.0 μL. The optimal volume of GNPs (10 nM) for all the reactions was 0.5 μL. The detection limits of DPO-nanoPCR for pMD19-T-VP6, pMD19-T-VP2, and pMD19-T-5′UTR were 9.40 × 10^2^ copies/μL, 5.14 × 10^3^ copies/μL, and 4.09 × 10^1^ copies/μL, respectively; and those using conventional PCR were 9.40 × 10^4^ copies/μL, 5.14 × 10^5^ copies/μL, and 4.09 × 10^4^ copies/μL, respectively. The sensitivity of DPO-nanoPCR was at least 100-fold higher than that of conventional PCR. The specificity detection showed that the DPO-nanoPCR was able to specifically detect BRV, BPV, and BVDV. Use of clinical samples indicated that target viruses can be detected accurately. Thus, DPO-nanoPCR is a new powerful, simple, specific, and sensitive tool for detecting mixed infections of BRV, BPV, and BVDV.

## Introduction

Bovine rotavirus (BRV) belongs to the genus *Rotavirus* in the family *Reoviridae* (Swiatek et al., 2010). BRV infection primarily occurs in calves between 15 and 45 days of age. The clinical symptoms include depression, loss of appetite, diarrhea, and dehydration. The rotavirus particle core has 11 double-stranded RNA segments (Crawford et al., 2017). Each segment encodes a different protein. The six structural proteins that have been identified are: VP1, VP2, VP3, VP4, VP6, and VP7. VP6 accounts for ∼51% of the total viral protein content. VP6 from group A rotavirus is highly conserved between the different serotypes with >90% amino acid sequence homology that enables it to be the major diagnostic antigen (Dennehy, 2015; Shepherd et al., 2018).

Bovine parvovirus (BPV) is a member of the *Bocaparvovirus* genus in the *Parvoviridae* family (Qiu et al., 2017). BPV infection mainly causes reproductive dysfunction in pregnant cows and respiratory and gastrointestinal diseases in newborn calves. The genome of BPV consists of three open reading frames (ORF): ORF1 encodes the non-structural protein NS1; ORF2 encodes the phosphorylated protein NP1; and ORF3 encodes the structural proteins VP1, VP2, and VP3. VP2 is the main structural protein of BPV that accounts for ∼80% of the total structural protein content (Dudleenamjil et al., 2010; Luo et al., 2013; Kailasan et al., 2015).

Bovine viral diarrhea virus (BVDV) is a globally well-distributed pathogen that infects cows leading to great economic losses (Yarnall and Thrusfield, 2017; Reichel et al., 2018). BVDV belongs to the *Pestivirus* genus under family *Flaviviridae* (Charoenlarp et al., 2018; Quintero Barbosa et al., 2019). In addition to causing respiratory, gastroenteric, and reproductive diseases, intrauterine infection with BVDV can also result in a persistent infection, thereby generating a state of immunotolerance (Lanyon et al., 2014; Khodakaram-Tafti and Farjanikish, 2017). The genome length of BVDV is ∼12.3 kb and consists of a 5′ UTR, ORF, and 3′ UTR. The 5′ UTR sequence is highly conserved in various strains of BVDV and is often used as a marker for diagnosis or classification of BVDV (Chernick and Frank, 2017; Yeşilbaǧ et al., 2017; Zoccola et al., 2017).

Bovine rotavirus, bovine parvovirus, and bovine viral diarrhea virus all cause intestinal infections. Owing to the similarity of their clinical manifestations and infection routes, mixed infections often occur. It is necessary to develop a method that simultaneously detects the three pathogens, which can save time and labor and has a huge advantage in clinical detection.

Dual-priming oligonucleotide primers were first proposed in 2007. DPO-based PCR is practical, reliable, and quick in detecting pathogens (Chun et al., 2007). The DPO primers comprise two separate initiation regions – a longer 5′ end and a shorter 3′ end (stabilizer and determinant) joined by a polyhypoxanthine (poly I) linker. Due to the special structure, DPO primers are difficult to form secondary structures. The 3′ end (6–12 base pairs long with a 40–80% GC-content) determines the specific extension of the target sequence and blocks subsequent false positive results (Kommedal et al., 2012; Ito and Suzaki, 2017). Studies have shown that mismatches of 3 or more bases in the 5′ and 3′ regions of the primer will not allow template extension. While the 5′ end (18–25 base pairs long with Tm >65°C) enables the use of a wide range of annealing temperatures (Lee H. R. et al., 2010), it is not necessary to screen primers, and optimize annealing temperatures (Lee H. J. et al., 2010). Therefore, DPO primers are perfect for developing multiplex PCR assays that can be used for amplifying multiple genes at multiple annealing temperatures (Yeh et al., 2011; Xu et al., 2015).

Nanoparticle-assisted PCR (nanoPCR) is an advanced PCR technique in which solid gold nanoparticles (GNPs) (1–100 nm) form a colloidal nanofluid to increase thermal conductivity and rapidly attain the target temperature (Li H. K. et al., 2005; Li M. et al., 2005; Rehman et al., 2015). The efficiency and sensibility of this assay are improved by shortening the time of amplification at non-target temperatures. The susceptibility and latency of BRV, BPV, and BVDV can lead to persistent infections in cows; and, the potential risks of shedding and dispersal lead to serious economic losses in the cattle industry. NanoPCR is particularly suitable for detecting samples with low viral titers in early and latent infection, which is of great significance for the prevention and control of diseases caused by BRV, BPV, and BVDV.

In this study, we combined DPO primers with nanoPCR to develop a multiplex DPO-nanoPCR system for the simultaneous detection of BRV, BPV, and BVDV. Compared to conventional PCR, DPO-nanoPCR saves time and effort and is very sensitive and specific. This is a new approach for diagnosing early and latent infections of BRV, BPV, and BVDV.

## Materials and Methods

### DPO Primer Design and Preparation of Recombinant Plasmids

Genes VP6 from BRV (NCDV strain), VP2 from BPV (ATCC strain VR-767) and 5′ UTR gene from BVDV (BA strain) were chosen based on the sequences in the GenBank database (GenBank accession numbers for VP6, VP2, and 5′ UTR are JF693031.1, NC_001540.1, and KC695814.1, respectively). The primers for VP6 (1,172 bp), VP2 (2,022 bp), and 5′ UTR (235 bp) were designed using the Oligo6.0 software (Table 1). VP6, VP2, and 5′ UTR were amplified and inserted into the pMD19-T vector (TaKaRa Bio Inc., Dalian, China) using standard cloning procedures. The recombinant plasmids were transformed into *E. coli* TG1. The plasmids were purified using the TIANprep Mini Plasmid Kit (TIANGEN Biotech, Beijing, China) and stored at −20°C.

**TABLE 1:** Primers used for DPO-nanoPCR and conventional PCR.

**Primer type**	**Gene**	**Name**	**Primer sequences (5′→3′)**	**Product size**
Normal	VP6	BRV-F	TTTCCCTTATTCAGCTTCATTCACGTTGAACAGATCGCA	450 bp
		BRV-R	AACGCCGCTACCGCTGGTGTCATATTTGGTGGTCTCATC	
DPO	VP6	BRV-DPOF	TTTCCCTTATTCAGCTTCATTCACIIIIIACAGATCGCA	450 bp
		BRV-DPOR	AACGCCGCTACCGCTGGTGTCATAIIIIITGGTCTCATC	
Normal	VP2	BPV-F	AGCGAGAACATTGTGGTCACTAAAAACACTCGCCAGTTTA	325 bp
		BPV-R	AGATGTGCATGCCTGCAGTCAGATCATTGTTGTAGACGG	
DPO	VP2	BPV-DPOF	AGCGAGAACATTGTGGTCACTAAAAIIIIICGCCAGTTTA	325 bp
		BPV-DPOR	AGATGTGCATGCCTGCAGTCAGATIIIIITTGTAGACGG	
Normal	5′UTR	BVDV-F	GTTGGATGGCTGAAGCCCTGAGTACAGGGTAGTCGTCA	180 bp
		BVDV-R	TGCAGCACCCTATCAGGCTGTATTCGTAGCGGTTGGTTA	
DPO	5′UTR	BVDV-DPOF	GTTGGATGGCTGAAGCCCTGAGTIIIIIGTAGTCGTCA	180 bp
		BVDV-DPOR	TGCAGCACCCTATCAGGCTGTATTIIIIICGGTTGGTTA	

Dual-priming oligonucleotides are composed of two unequal regions (a long 5′-segment and a short 3′-segment) linked with 5 poly (I) stretches (Chun et al., 2007). The primers for DPO-nanoPCR and conventional PCR were designed using the Oligo6.0 software based on the conserved regions of VP6, VP2, and 5′ UTR (Table 2) and the amplicon sizes were 450 bp, 325 bp, and 180 bp, respectively.

**TABLE 2:** Primers used for preparation of the recombinant plasmids.

**Plasmid**	**Name**	**Primer Sequences (5′→3′)**	**Product size**
pMD-19T-VP6	BRV-VP6-F	ATGGATGTCCTGTACTCCTTGTC	1172 bp
	BRV-VP6-R	ATGGAAGCCACTGTAAATACACG	
pMD-19T-VP2	BPV-VP2-F	ATGCCGCCAACCAATAAAGCTAATT	2022 bp
	BPV-VP2-R	CTACAGGACTTTGTGGTGATTGAATC	
pMD-19T-5′UTR	BVDV-5′UTR-F	AAACAAGGAGGGTAGCAACAGTG	235 bp
	BVDV-5′UTR-R	TTTAGTAGCGATACAGTGGGCCT	

### Sample Collection and RNA/DNA Isolation

We collected 269 fecal samples from the Heilongjiang, Jilin, Liaoning, and Inner Mongolia provinces. BRV (strain NCDV), BPV (ATCC strain VR-767), BVDV (strain BA), bovine respiratory syncytial virus (BRSV; strain 391-2), PoRV (strain JL94), PPV (strain TJ), TGEV (strain TH98), and PEDV (strain LJB/03) were stored in our lab. BRV (strain NCDV), BPV (ATCC strain VR-767), and BVDV (strain BA) were propagated using the MA-104, BT, and MDBK cell lines, respectively.

We extracted nucleic acids from the viruses using magnetic beads (PuriMag Biotechnology Ltd., Xiamen, China) with modifications of previously described protocols (Smerkova et al., 2013; Clark et al., 2019; Li et al., 2019; Oberacker et al., 2019). First, 500 μL of phosphate-buffered saline was added to 0.2 g of feces in a centrifuge tube, shaken for 2 min, and frozen and thawed three times. After centrifugation at 12,000 r/min for 10 min, 250 μL of the supernatant was transferred into an EP tube. 500 μL of the lysis buffer [4 M guanidinium thiocyanate, 0.5 M Tris–HCl, 0.015 M sodium citrate, 5% (w/v) sodium dodecyl sulfate, and 0.1 M EDTANa_2_] was then added and mixed for 2 min. The mixture was incubated on ice for 10 min and shaken for 1 min followed by the addition of 20 μL of the magnetic bead suspension and mixed. The mixture was incubated at 4°C for 10 min and placed on a magnetic frame for 5 min. The aqueous phase was carefully transferred to a new EP tube following which an equal volume of cold isopropanol was added, mixed, and the mixture was incubated at −20°C for 2 h and placed on a magnetic frame for 5 min. The supernatant was discarded and 1 mL of cold 75% ethanol was subsequently added. After one wash, the tubes were placed on a magnetic frame and incubated for 5 min. The supernatants were discarded and samples were air-dried. Subsequently, 50 μL of diethylpyrocarbonate-treated water was added to dissolve the DNA/RNA pellet. The TransScript Fly First-Strand cDNA Synthesis SuperMix kit (TransGen Biotech Co., Beijing, China) was used to synthesize cDNA from the samples. The DNA and cDNA samples were stored at −20°C.

### Preparation and Characterization of Gold Nanoparticles (GNPs)

Gold nanoparticles with an average diameter of 10 nm were synthesized using the Turkevich and Frens synthesis method (Emmanuel et al., 2018). One hundred milliliters of 0.01% gold chloride was boiled for 3 min following which 5 mL of 1% trisodium citrate solution (preheated to 37°C) was rapidly added while stirring with a glass rod. The solution was boiled again for 5–8 min until it turned wine red. The GNPs were visualized using a transmission electron microscope after cooling at room temperature and then stored at 4°C.

### Optimization of the Conditions for DPO-nanoPCR Assay

We optimized the annealing temperature, primer concentration, and GNP concentration for the DPO-nanoPCR assay. The recombinant plasmids (pMD19-T-VP6, pMD19-T-VP2, and pMD19-T-5′ UTR) were used as templates. Conventional PCR primers usually require a specific annealing temperature. Theoretically, DPO primers are very specific over a wide range of annealing temperatures owing to their structural features (Chun et al., 2007; Xu et al., 2017). Therefore, different annealing temperatures were used to verify whether the detection can be affected with varying annealing temperatures. We used temperatures between 41 and 65°C that were chosen randomly. The reactions were performed at different annealing temperatures using DPO primers, and conventional primers were used as controls. Since reagents are limited in the multiple PCR amplification system, each pair of primers compete for the reagents in the reaction. To enhance amplification, the volume and ratios of the three pairs of primers need to be optimized (Luo et al., 2015; Wang et al., 2015). The volumes of all the primers (10 μM) used ranged from 0.1 to 1.0 μL in increments of 0.1 μL. To test the effects of GNPs on amplification, the volumes of GNPs (10 nM) used were between 0.1 and 1.0 μL in increments of 0.1 μL.

The reaction conditions were as follows: 95°C for 5 min followed by 30 cycles of 94°C for 30 s, 41–65°C for 30 s, and 72°C for 30 s and a final extension at 72°C for 10 min. Products were visualized on 2% agarose gels.

### Analyzing the Sensitivity and Reproducibility of DPO-nanoPCR

To analyze the sensitivity of DPO-nanoPCR, the pMD19-T-VP6, pMD19-T-VP2, and pMD19-T-5′ UTR plasmids were purified using the TIANprep Mini Plasmid Kit (TIANGEN Biotech, Beijing, China) and quantified by UV spectroscopy (Thermo Scientific NanoDrop 2000 Spectrophotometer, Thermo Fisher Scientific, United States). Plasmid copy number (copies/μL) was calculated according to the following equation: [6.02 × 10^23^ (copy/mol) × DNA amount (g) × 10^–9^)/(DNA length (dp) × 660 (g/mol/dp)] (Kang et al., 2018). Ten-fold serial dilutions of the recombinant plasmids were used to analyze the sensitivity of DPO-nanoPCR compared to conventional PCR. Conventional PCRs were performed using the same primers and reaction conditions. The amplicons were analyzed by 2% agarose gel electrophoresis. All experiments were repeated and validated by multiple trials.

The reproducibility of DPO-nanoPCR was determined using three different concentrations of standard plasmid. Each dilution was analyzed in three independent experiments performed by two different operators on different days in accordance with MIQE guidelines (Bustin et al., 2009).

### Analyzing the Specificity of DPO-nanoPCR

DNA and cDNA samples from BRV, BPV, BVDV, BRSV, PoRV, PPV, TGEV, and PEDV were used to assess the specificity of DPO-nanoPCR. A mixture of the BRV, BPV, and BVDV cultures was used as a positive control. The amplicons were analyzed on a 2% agarose gel.

### Detection of Clinical Samples

A total of 269 clinical samples were tested by the DPO-nanoPCR developed in this study. These results were compared with those from the conventional PCR assay using 2% agarose gel electrophoresis.

### DNA Sequencing

The DPO-nanoPCR amplicons were sent to Kumei Company (Changchun, China) for sequencing. The sequences obtained were confirmed by the DNAStar software and BLAST of GenBank.

## Results

### Characterization of the Synthesized Gold Nanoparticles

**FIGURE 1:**
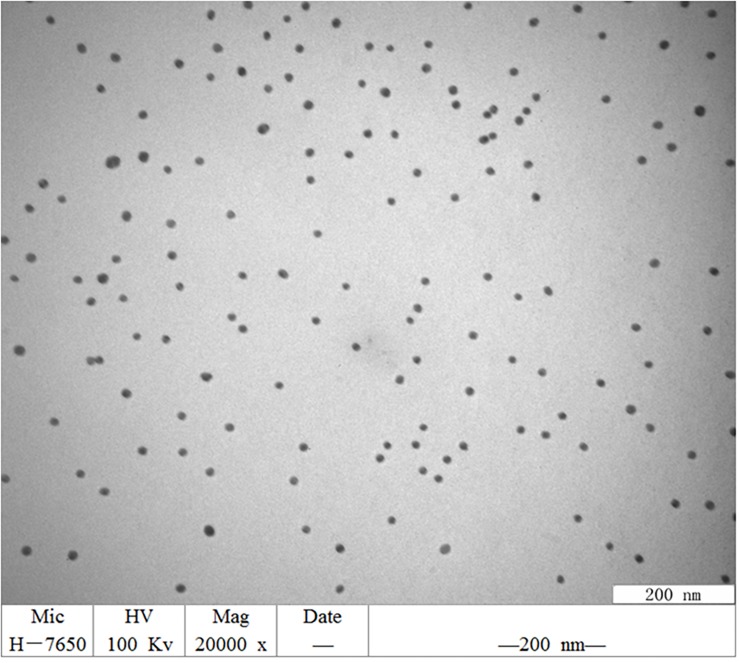
TEM image of GNPs synthesized. GNPs had a relatively regular size and spherical morphology, uniform particle size, no impurity fragments, and no agglutination.

The synthesized GNPs appeared wine red. Transmission electron micrographs showed that these GNPs were relatively regular in size and spherical morphology and had uniform particle size without impurities and agglutination (Figure 1).

### Optimizing the DPO-nanoPCR Assay

**FIGURE 2:**
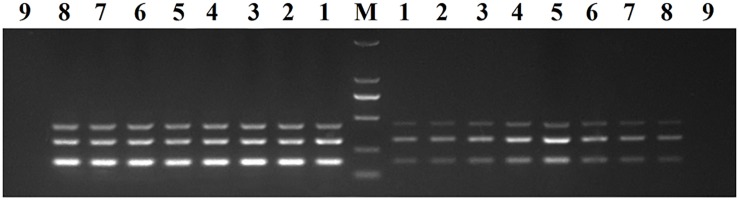
Optimization of the annealing temperature. The results of DPO-nanoPCR were on the left and the results of conventional PCR were on the right. Lane M, DL2000 DNA marker; Lane 1–8, 41.7°C, 45.6°C, 48.8°C, 52.6°C, 56.2°C, 60.9°C, 63.4°C, and 64.1°C; Lane 9, negative control.

**FIGURE 3:**
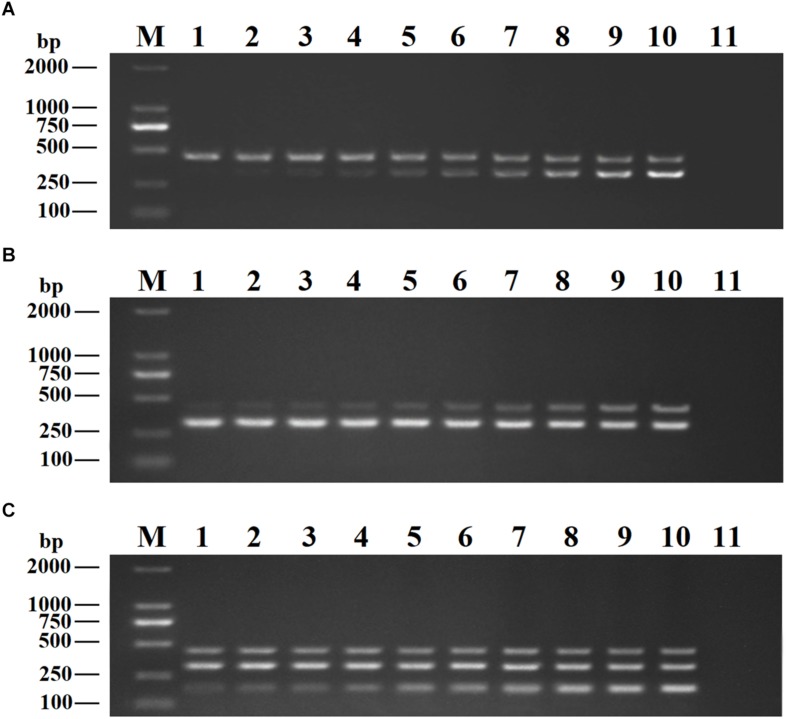
Optimization of primer concentrations. The recombinant plasmids (pMD19-T-VP6, pMD19-T-VP2, and pMD19-T-5′UTR) were used as templates. **(A)** When the volumes of BRV-DPOF (10 μM) and BRV-DPOR (10 μM) were 1.0 μL, the volumes of BPV-DPOF (10 μM), and BPV-DPOR (10 μM) were tested ranging from 0.1 to 1.0 μL in increments of 0.1 μL. **(B)** When the volumes of BPV-DPOF (10 μM) and BPV-DPOR (10 μM) were 1.0 μL, the volumes of BRV-DPOF (10 μM), and BRV-DPOR (10 μM) were tested ranging from 0.1 to 1.0 μL in increments of 0.1 μL. **(C)** When the volumes of BRV-DPOF (10 μM), BRV-DPOR (10 μM), BPV-DPOF (10 μM), and BPV-DPOR (10 μM) were all 1.0 μL, the volumes of BVDV-DPOF (10 μM) and BVDV-DPOR (10 μM) were tested ranging from 0.1 to 1.0 μL in increments of 0.1 μL. Lane M, DL2000 DNA maker; Lane 1–10, 0.1 μL, 0.2 μL, 0.3 μL, 0.4 μL, 0.5 μL, 0.6 μL, 0.7 μL, 0.8 μL, 0.9 μL and 1.0 μL, respectively; Lane 11, negative control.

**FIGURE 4:**
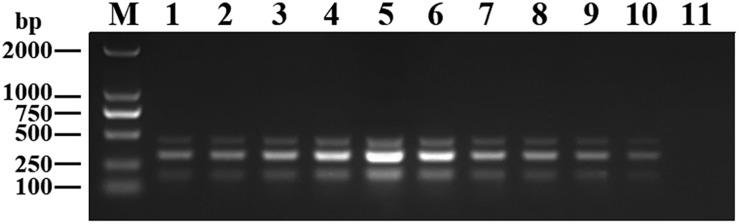
Optimization of GNPs concentration. The volumes of GNPs (10 nM) were tested ranging from 0.1 to 1.0 μL in increments of 0.1 μL. Lane M, DL2000 DNA maker; Lane 1–10, 0.1 μL, 0.2 μL, 0.3 μL, 0.4 μL, 0.5 μL, 0.6 μL, 0.7 μL, 0.8 μL, 0.9 μL, and 1.0 μL, respectively; Lane 11, negative control.

Recombinant plasmids (pMD19-T-VP6, pMD19-T-VP2, and pMD19-T-5′ UTR) were used as templates to optimize the DPO-based nanoPCR assay. The annealing temperature used ranged between 41 and 65°C. Figure 2 shows that PCR using DPO primers could efficiently amplify the target under conditions of varying annealing temperatures. However, PCR amplification was most efficient at 56.2°C using conventional primers. As shown in Figures 3, 4, the optimal volume of all the primers (10 μM) was 1.0 μL and that of the GNPs (10 nM) for all the reactions was 0.5 μL.

The reaction conditions of DPO-nanoPCR for detecting BRV, BPV, and BVDV were optimized. The DPO-nanoPCR reaction system (25 μL) comprised: 1 μL of cDNA, 0.5 μL of ExTaq (5 U/μL; TaKaRa, Dalian, China), 5 μL of 10 × ExTaq PCR Buffer (with Mg^2+^; 20 mM), 10 μM of the forward and reverse DPO primers each, 2.5 μL of dNTPs (2.5 mM), and 0.5 μL of the GNPs (10 nM). Nuclease-free water was used to make the volume up to 25 μL. The reaction conditions of DPO-nanoPCR were as follows: 95°C for 5 min followed by 30 cycles of 94°C for 30 s, 55°C for 30 s, and 72°C for 30 s and a final extension at 72°C for 10 min. The amplicons were visualized using 2% agarose gels.

**FIGURE 5:**
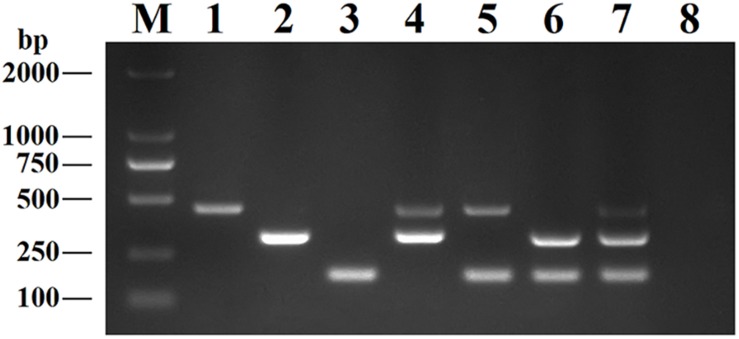
The DPO-nanoPCR assay for detection of BRV, BPV and BVDV. Lane M: DL2000 DNA marker; Lane 1, pMD19-T-VP6; Lane 2, pMD19-T-VP2; Lane 3, pMD19-T-5′UTR; Lane 4, pMD19-T-VP6 and pMD19-T-VP2; Lane 5, pMD19-T-VP6 and pMD19-T-5′UTR; Lane 6, pMD19-T-VP2 and pMD19-T-5′UTR; Lane 7, pMD19-T-VP6, pMD19-T-VP2 and pMD19-T-5′UTR; Lane 8, negative control.

This DPO-nanoPCR assay was developed for simultaneously detecting BRV, BPV, and BVDV based on the optimized reaction system. Figure 5 shows that bands obtained by the optimized DPO-nanoPCR were clear and specific (with a clean negative control).

### Sensitivity and Reproducibility of the DPO-nanoPCR Assay

**FIGURE 6:**
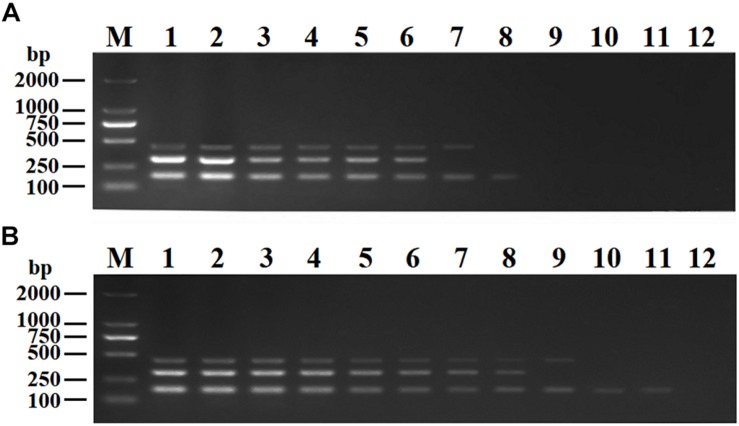
Sensitivity of conventional PCR **(A)** and DPO-nanoPCR **(B)**. A serial 10-fold diluted plasmid mixture was used. Lane M, DL2000 DNA marker; Lane 1–11, pMD19-T-VP6 concentrations ranging from 9.40 × 10^10^copies/μL to 9.40 × 10^0^ copies/μL, pMD19-T-VP2 concentrations ranging from 5.14 × 10^10^ copies/μL to 5.14 × 10^0^ copies/μL, and pMD19-T-5′UTR concentrations ranging from 4.09 × 10^11^ to 4.09 × 10^1^ copies/μL; Lane 12, negative control.

The purified pMD19-T-VP6, pMD19-T-VP2, and pMD19-T-5′ UTR plasmids were quantified using UV spectroscopy (Thermo Scientific NanoDrop 2000 Spectrophotometer; Thermo Fisher Scientific, United States). Ten-fold serial dilutions of the recombinant plasmids (9.40 × 10^10^ copies/μL of pMD19-T-VP6, 5.14 × 10^10^ copies/μL of pMD19-T-VP2, and 4.09 × 10^11^ copies/μL of pMD19-T-5′ UTR) were used to determine the sensitivity of the DPO-nanoPCR assay. The results indicated that the detection limits for pMD19-T-VP6, pMD19-T-VP2, and pMD19-T-5′ UTR were 9.40 × 10^2^ copies/μL, 5.14 × 10^3^ copies/μL, and 4.09 × 10^1^ copies/μL, respectively (Figure 6B) and those using conventional PCR were 9.40 × 10^4^ copies/μL, 5.14 × 10^5^ copies/μL, and 4.09 × 10^4^ copies/μL (Figure 6A), respectively. Thus, the sensitivity of DPO-nanoPCR was at least 100-fold higher than that of conventional PCR. The reproducibility of DPO-nanoPCR assay was evaluated by testing different concentrations of standard plasmids. Detection results were identical (Supplementary Figure S1). The results indicated satisfied reproducibility for DPO-nanoPCR assay.

### Specificity of the DPO-nanoPCR Assay

**FIGURE 7:**
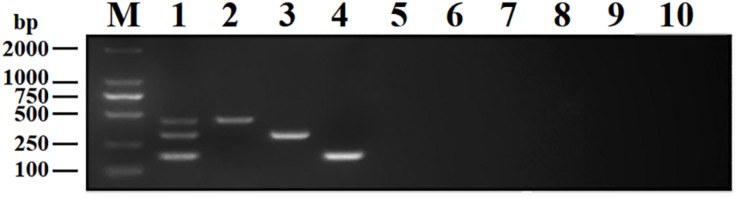
Specificity of the DPO-nanoPCR assay for detecting BRV, BPV, and BVDV. Lane M, DL2000 DNA marker; Lane 1, BRV, BPV, and BVDV; Lane 2, BRV; Lane 3, BPV; Lane 4, BVDV; Lane 5, BRSV; Lane 6, PoRV; Lane 7, PPV; Lane 8, PEDV; Lane 9, TGEV; Lane 10, negative control.

To analyze the specificity of DPO-nanoPCR, DNA or cDNA samples from BRV, BPV, BVDV, BRSV, PoRV, PPV, TGEV, and PEDV were used. The results showed that DPO-nanoPCR could not amplify any of the other five viruses except BRV, BPV, and BVDV (Figure 7), indicating that the assay is specific.

### Detection of Clinical Samples

DPO-nanoPCR and conventional PCR were used to test 269 clinical samples. The results are shown in Table 3. Thirteen (4.8%), fourteen (5.2%), and twenty-one samples (7.8%) were positive for BRV, BPV, and BVDV, respectively using DPO-nanoPCR. Ten (3.7%), nine (3.3%), and sixteen samples (5.9%) were positive for BRV, BPV, and BVDV, respectively, using conventional PCR. Of all the clinical samples, 2.6% (7 out of 269) were positive for BRV, BPV, and BVDV by DPO-nanoPCR, and 0.7% (2 out of 269) were positive for BRV, BPV, and BVDV by conventional PCR (Table 3).

**TABLE 3:** Detection of clinical samples by DPO-nanoPCR and conventional PCR.

**Sample source**	**Number of samples**	**DPO-nanoPCR**				**Conventional PCR**			
			
		**Ratio of BRV positive samples**	**Ratio of BPV positive samples**	**Ratio of BVDV positive samples**	**Ratio of BRV, BPV, and BVDV positive samples**	**Ratio of BRV positive samples**	**Ratio of BPV positive samples**	**Ratio of BVDV positive samples**	**Ratio of BRV, BPV, and BVDV positive samples**
Heilongjiang	94	4/94	6/94	8/94	2/94	4/94	2/94	7/94	1/94
Jilin	53	3/53	2/53	2/53	1/53	1/53	2/53	2/53	0/53
Liaoning	21	1/21	3/21	1/21	1/21	1/21	2/21	1/21	0/21
Neimenggu	101	5/101	3/101	10/101	3/101	4/101	3/101	6/101	1/101
Total	269	13/269	14/269	21/269	7/269	10/269	9/269	16/269	2/269

Positive results obtained by conventional PCR were consistent with DPO-nanoPCR. Three samples were positive for BRV by DPO-nanoPCR but negative by conventional PCR. Five samples were positive for BPV by DPO-nanoPCR but negative by conventional PCR. Three samples were positive for BVDV by DPO-nanoPCR but negative by conventional PCR. However, there were no samples that were found to be negative using DPO-nanoPCR but positive using conventional PCR. Sequence analysis showed high similarity (100%) between the reference sequences of the target viruses and the DPO-nanoPCR amplicons. All these results indicated that DPO-nanoPCR assay is more sensitive than conventional PCR.

## Discussion

With the advances in the cattle industry, large-scale and intensive breeding has led to increased physical contact between cattle. This often enables and accelerates the spread of contagious diseases resulting in mixed infections of two or more pathogens. BRV, BPV, and BVDV are all transmitted by the fecal-oral route and shed through feces. The clinical symptoms of BRV, BPV, and BVDV infections are similar. They all primarily infect newborn calves and cause diarrhea (Rigo-Adrover et al., 2019). Mixed infections cause severe diarrhea that leads to increased mortality (Ruiz et al., 2015; Fan et al., 2017) and make clinical diagnosis difficult. Therefore, it is imperative to develop a diagnostic method that can simultaneously detect multiple pathogens resulting in enhanced epidemic surveillance. In this study, we combined DPO primers with a nanoPCR assay to establish a multiplex DPO-nanoPCR method for the simultaneous detection of BRV, BPV, and BVDV. To the best of our knowledge, this is the first report of a detection method combining DPO primers with nanoPCR.

Multiplex PCR is a rapid and economical assay that is often used for the detection of mixed infections. However, conventional primer system-based multiplex PCR using multi-primer sets often shows a lower specificity of amplification owing to primer competition, formation of primer dimers, or the different annealing temperatures used (Chun et al., 2007). DPOs have two separate primer segments – one of which is longer than the other – joined by a polydeoxyinosine [poly (I)] linker. Since DPO primers have special structures, non-specific hybridization between primers, and nucleotide sequences is prevented such that non-specific amplification can be eliminated without disrupting the amplification of target sequences (Chun et al., 2007; Fu et al., 2017; Xu et al., 2017).

We used the DPO-nanoPCR assay to detect the presence of BRV, BPV, BVDV, and other viruses. Only the target viruses were specifically detected and the presence of no other viruses were observed. Moreover, the DPO primers were not sensitive to changes in the annealing temperature. Using the 41–65°C range of annealing temperatures, there was specific amplification of the target genes. The specificity of detection was not affected upon altering the annealing temperatures within a certain range. So far, various PCR methods using DPO primers have been widely used in multiplex PCR, genotyping PCR, real-time PCR, and reverse transcription PCR for the detection of bacterial and viral pathogens (Chung et al., 2014; Xu et al., 2017). It has been reported that DPO-based PCR showed higher specificity for target sequences compared to conventional PCR (Ma et al., 2015; Chung et al., 2016). Taken together, these findings prove that DPO-based PCR is a reliable method for clinical diagnosis.

In this study, efficiency of the DPO-PCR assay was improved using nanoparticles. Nanofluids are formed upon the addition of GNPs into the PCR system. Nanofluids possess greater thermal conductivity. Thus, thermal conductivity is enhanced in nanoparticle-containing PCR systems that enables attaining the target temperature in a shorter time. Efficient heat transfer generates a larger number of amplicons, thereby improving the sensitivity of the reaction (Gabriel et al., 2018). We developed a multiplex DPO-nanoPCR assay for detecting BRV, BPV, and BVDV that is 100–1000 times more sensitive than conventional multiplex PCR. This indicates that the GNPs increase productivity by acting as modulators of PCR. Using the multiplex DPO-nanoPCR method to detect BRV, BPV, and BVDV in clinical samples showed that the target viruses could be specifically detected, and the DPO-nanoPCR assay was more sensitive than conventional PCR. It has also been reported that nanoRT-PCR exhibits a 10–100-fold higher sensitivity than conventional RT-PCR. Ma et al. (2013) established a nanoPCR method to rapidly detect and distinguish between the field and vaccine strains of the pseudorabies virus. The sensitivity of this method was 100–1000 times higher than that of conventional PCR (Ma et al., 2013). Liu et al. (2019) developed a nanoPCR assay for detecting BRSV. The sensitivity of this assay was also 10 times higher than that of conventional PCR (Liu et al., 2019). These studies prove that the addition of GNPs to PCRs effectively increase the sensitivity of PCR.

To the best of our knowledge, this is the first report of using a combination of nanoPCR with DPOs for simultaneously detecting BRV, BPV, and BVDV. Detection using DPO-nanoPCR was 100–1000 times more sensitive than conventional multiplex PCR. Thus, this DPO-nanoPCR assay is a new powerful tool that has great potential in clinical diagnoses of BRV, BPV, and BVDV.

## Data Availability Statement

Nucleotide sequences generated for this study can be found in the NCBI GenBank, MN565845, MN565846, MN565847, MN565848, MN565849, MN565850, MN565851, MN565852, MN565853, MN565854, MN565855, MN565856, MN565857, MN567095, MN567096, MN567097, MN567098, MN567099, MN567100, MN567101, MN567102, MN567103, MN567104, MN567105, MN567106, MN567107, MN567108, MN565858, MN565859, MN565860, MN565861, MN565862, MN565863, MN565864, MN565865, MN565866, MN565867, MN565868, MN565869, MN565870, MN565871, MN565872, MN565873, MN565874, MN565875, MN565876, MN565877, and MN565878.

## Ethics Statement

This study was carried out in accordance with the principles of the Basel Declaration and recommendations of Guidelines on Animal Experimentation, the Ethical Committee for Animal Sciences of Heilongjiang Province. The protocol was approved by the Ethical Committee for Animal Sciences of Heilongjiang Province.

## Author Contributions

MW and YY developed the DPO-nanoPCR assay. YJ synthesized the gold nanoparticles. RW and LW optimized the reaction conditions. HZ collected the clinical samples. WC detected the clinical samples. XQ, YL, YX, and LT conceived the project. XQ was the grant holder and drafted the manuscript. All authors read, revised, and approved the final manuscript.

## Conflict of Interest

The authors declare that the research was conducted in the absence of any commercial or financial relationships that could be construed as a potential conflict of interest.

## Supplementary Material

FIGURE S1 Reproducibility test results. The reproducibility of DPO-nanoPCR assay was evaluated by testing different concentrations of standard plasmids. Each dilution was analyzed in three independent experiments performed by two different operators **(A,B)**. A serial 10-fold diluted plasmid mixture was used. Lane M, DL2000 DNA marker. **(A1,B1)** Lane 1–11, pMD19-T-VP6 concentrations ranging from 9.40 × 10^10^ copies/μL to 9.40 × 10^0^ copies/μL, pMD19-T-VP2 concentrations ranging from 5.14 × 10^10^ copies/μL to 5.14 × 10^0^ copies/μL, and pMD19-T-5′UTR concentrations ranging from 4.09 × 10^11^ to 4.09 × 10^1^ copies/μL. Lane 12, negative control. **(A2,B2)** Lane 1–11, pMD19-T-VP6 concentrations ranging from 9.40 × 10^9^ copies/μL to 9.40 × 10^–1^ copies/μL, pMD19-T-VP2 concentrations ranging from 5.14 × 10^9^ copies/μL to 5.14 × 10^–1^ copies/μL, and pMD19-T-5′UTR concentrations ranging from 4.09 × 10^10^ to 4.09 × 10^0^ copies/μL. Lane 12, negative control. **(A3,B3)** Lane 1–11, pMD19-T-VP6 concentrations ranging from 9.40 × 10^8^ copies/μL to 9.40 × 10^–2^ copies/μL, pMD19-T-VP2 concentrations ranging from 5.14 × 10^8^ copies/μL to 5.14 × 10^–2^ copies/μL, and pMD19-T-5′UTR concentrations ranging from 4.09 × 10^9^ to 4.09 × 10^–1^ copies/μL. Lane 12, negative control.
