# Supplementary figures and images for: Simultaneous Detection of Bovine Rotavirus, Bovine Parvovirus, and Bovine Viral Diarrhea Virus Using a Gold Nanoparticle-Assisted PCR Assay With a Dual-Priming Oligonucleotide System
Source: Front Microbiol. 2019 Dec 12;10:2884. doi: 10.3389/fmicb.2019.02884 (PMC6920155; doi:10.3389/fmicb.2019.02884)

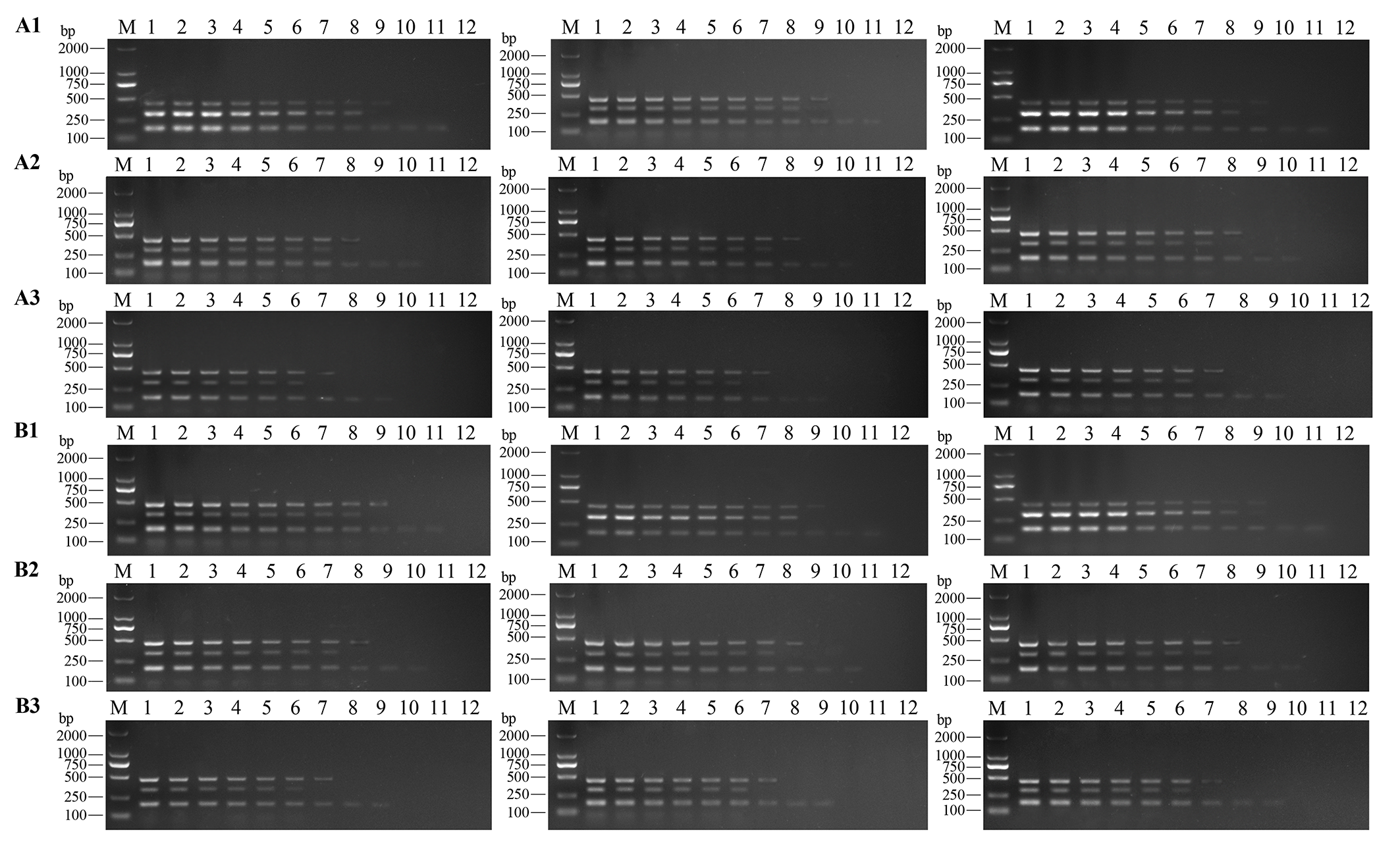

Supplement: FIGURE S1 — Reproducibility test results. The reproducibility of DPO-nanoPCR assay was evaluated by testing different concentrations of standard plasmids. Each dilution was analyzed in three independent experiments performed by two different operators (A,B). A serial 10-fold diluted plasmid mixture was used. Lane M, DL2000 DNA marker. (A1,B1) Lane 1–11, pMD19-T-VP6 concentrations ranging from 9.40 × 10^10^ copies/μL to 9.40 × 100 copies/μL, pMD19-T-VP2 concentrations ranging from 5.14 × 10^10^ copies/μL to 5.14 × 100 copies/μL, and pMD19-T-5′UTR concentrations ranging from 4.09 × 10^11^ to 4.09 × 101 copies/μL. Lane 12, negative control. (A2,B2) Lane 1–11, pMD19-T-VP6 concentrations ranging from 9.40 × 10^9^ copies/μL to 9.40 × 10^–1^ copies/μL, pMD19-T-VP2 concentrations ranging from 5.14 × 10^9^ copies/μL to 5.14 × 10^–1^ copies/μL, and pMD19-T-5′UTR concentrations ranging from 4.09 × 10^10^ to 4.09 × 100 copies/μL. Lane 12, negative control. (A3,B3) Lane 1–11, pMD19-T-VP6 concentrations ranging from 9.40 × 10^8^ copies/μL to 9.40 × 10^–2^ copies/μL, pMD19-T-VP2 concentrations ranging from 5.14 × 10^8^ copies/μL to 5.14 × 10^–2^ copies/μL, and pMD19-T-5′UTR concentrations ranging from 4.09 × 10^9^ to 4.09 × 10^–1^ copies/μL. Lane 12, negative control. [file Image_1.TIF]
